# Structural characterization of the Sel1‐like repeat protein LceB from *Legionella pneumophila*

**DOI:** 10.1002/pro.4889

**Published:** 2024-03-01

**Authors:** Tiffany V. Penner, Neil Lorente Cobo, Deepak T. Patel, Dhruvin H. Patel, Alexei Savchenko, Ann Karen C. Brassinga, Gerd Prehna

**Affiliations:** ^1^ Department of Microbiology University of Manitoba Winnipeg Manitoba Canada; ^2^ Department of Microbiology, Immunology and Infectious Diseases University of Calgary Calgary Alberta Canada

**Keywords:** effector, LceB, *Legionella pneumophila*, Lpg1356, Sel1‐like repeat protein, Type IV secretion system, X‐ray crystallography

## Abstract

*Legionella* are freshwater Gram‐negative bacteria that in their normal environment infect protozoa. However, this adaptation also allows *Legionella* to infect human alveolar macrophages and cause pneumonia. Central to *Legionella* pathogenesis are more than 330 secreted effectors, of which there are nine core effectors that are conserved in all pathogenic species. Despite their importance, the biochemical function of several core effectors remains unclear. To address this, we have taken a structural approach to characterize the core effector of unknown function LceB, or Lpg1356, from *Legionella pneumophila.* Here, we solve an X‐ray crystal structure of LceB using an AlphaFold model for molecular replacement. The experimental structure shows that LceB adopts a Sel1‐like repeat (SLR) fold as predicted. However, the crystal structure captured multiple conformations of LceB, all of which differed from the AlphaFold model. A comparison of the predicted model and the experimental models suggests that LceB is highly flexible in solution. Additionally, the molecular analysis of LceB using its close structural homologs reveals sequence and structural motifs of known biochemical function. Specifically, LceB harbors a repeated KAAEQG motif that both stabilizes the SLR fold and is known to participate in protein–protein interactions with eukaryotic host proteins. We also observe that LceB forms several higher‐order oligomers in solution. Overall, our results have revealed that LceB has conformational flexibility, self‐associates, and contains a molecular surface for binding a target host‐cell protein. Additionally, our data provides structural insights into the SLR family of proteins that remain poorly studied.

## 1 INTRODUCTION

*Legionella* species are Gram‐negative bacteria ubiquitous in freshwater environments (Fliermans et al., 1981) where they parasitize protozoa (Rowbotham, 1980). By evolving with protozoa, *Legionella* has established survival and replication mechanisms to persist within eukaryotic cells such as amoeba. Specifically, the similarity of an amoeba to macrophages has primed *Legionella* to be a serious pathogen capable of infecting human alveolar macrophages (Park et al., 2020; Rowbotham, 1980). Infection of human alveolar macrophages can occur by the inhalation of contaminated water aerosols. This may result in Legionnaires' disease which is characterized by severe pneumonia, primarily in elderly and/or immunocompromised individuals (Davis et al., 1982; Fraser et al., 1977; Mondino et al., 2020; Nash et al., 1984; Viasus et al., 2022). Prompted by phagocytosis, *Legionellae* utilize the Dot/Icm Type IV secretion system (T4SS) to translocate effector proteins into the host cytosol to establish a replicative niche known as the *Legionella*‐containing vacuole (LCV) (Horwitz & Silverstein, 1980; Nagai & Roy, 2001). Vesicles from the smooth endoplasmic reticulum (ER) fuse with the LCV membrane and eventually become studded with ribosomes and mitochondria (Horwitz, 1983a). The LCV evades fusion with lysosomes and maintains a higher pH than vacuoles with formalin‐killed *Legionella pneumophila* (Horwitz, 1983b; Horwitz & Maxfield, 1984). To establish and maintain the LCV, T4SS effector proteins are secreted into the host macrophage which are crucial for manipulating host cell signaling pathways and virulence (Finsel & Hilbi, 2015; Von Dwingelo et al., 2019). In *L. pneumophila* alone, more than 330 effectors are known to be translocated (Mondino et al., 2020). Moreover, bioinformatic studies have predicted that there are over 18,000 T4SS effectors from 58 *Legionella* species, with only nine that are conserved in all species. As such, these nine proteins have been deemed the core effectors (Gomez‐Valero et al., 2019; Wexler et al., 2022). Given the vast array of effectors, the majority of these proteins have not been fully characterized due to host specificity and redundancy (Best & Kwaik, 2018; Mondino et al., 2020). Those T4SS proteins that have been studied, display a variety of functions including evasion from the endocytic maturation pathway, interaction with the ER, kinase signaling, epigenetic regulation, mRNA processing and manipulation of the ubiquitin pathway (Mondino et al., 2020).

As many *Legionella* effectors directly bind protein targets in their hosts, these effectors often contain tetratricopeptide repeats (TPRs). TPRs were first discovered in the eukaryote *Saccharomyces cerevisiae* gene product CDC23 which has roles in the synthesis of RNA and mitosis (Sikorski et al., 1990). These motifs are composed of 34 loosely conserved, alternating large and small sidechain residues. TPRs are usually found in proteins as 3–16 tandem repeats but may be dispersed throughout the protein (Letunic et al., 2021; Sikorski et al., 1990). In bacterial species, TPR proteins are known to function in several pathways including biomineralization of iron oxides in magnetotactic bacteria (Zeytuni et al., 2011), assembly of the outer membrane (Gatsos et al., 2008), natural competence (Neiditch et al., 2017; Talagas et al., 2016) and pathogenesis (Cerveny et al., 2013; Neiditch et al., 2017; Notti & Stebbins, 2016) including the movement of virulence factors into host cells (Bröms et al., 2006; Edqvist et al., 2006). Although TPR proteins participate in a wide variety of biological functions, one commonality is their importance in protein–protein interactions (D'Andrea & Regan, 2003; Lamb et al., 1995). Given this, TPRs are ubiquitous in nature (D'Andrea & Regan, 2003) and found in all three domains of life (Jernigan & Bordenstein, 2015).

A subtype of TPR proteins exist termed Sel1‐like repeat (SLR) proteins, which are a structural variation of the canonical TPR (Paysan‐Lafosse et al., 2023). Although the α‐helical conformations adopted by TPR and SLR proteins are similar, there are spatial differences between TPR and SLR proteins. This was first shown in the structure of the SLR protein HcpB from *Helicobacter pylori* (Luthy et al., 2002). As compared to TPRs, SLRs have 4–12 additional residues in the loop between the two α‐helices of a single repeat unit, but two fewer residues in the repeat connector (Mittl & Schneider‐Brachert, 2007). Furthermore, the SLR sequence is longer than the TPR as it consists of 36–44 amino acid residues (Mittl & Schneider‐Brachert, 2007). However, both motifs contain the characteristic pattern of large and small residue sidechains, including tyrosine. For example, according to the SMART database (Letunic et al., 2021) the canonical SLR motif is 3A‐7L‐8G‐11Y‐14G‐16G‐20D‐24A‐31A‐32A‐35G, and the canonical TPR motif is 4 W‐7L‐8G‐11Y‐20A‐24F‐27A‐32P (Sikorski et al., 1990).

SLR proteins were first described in the round‐worm *Caenorhabditis elegans* (Grant & Greenwald, 1996). In this work, the extracellular SEL‐1 protein was shown to be a negative regulator of membrane receptor proteins Lin‐12 and Glp‐1 which are both important for determining the fate of a cell (Grant & Greenwald, 1996). Although the exact mechanism of SEL‐1 is unknown, it is likely that SEL‐1 targets the Lin‐12 receptor when bound to a ligand for subsequent degradation (Grant & Greenwald, 1997). Bacterial SLRs have been shown to function in exopolysaccharide synthesis in Pseudomonas (Jain & Ohman, 1998), flagellar motility in *Vibrio parahemolyticus* (McCarter, 1994) and infection in *L. pneumophila* by mediating the interaction between the bacteria and its eukaryotic host (Bröms et al., 2006; Cirillo et al., 2000; Newton et al., 2006) Not unlike TPR proteins, SLR proteins have a variety of functions that are part of signal transduction pathways and immunomodulatory functions (Mittl & Schneider‐Brachert, 2007). However, limited literature exists on SLR proteins as compared to TPR domains due to the lower abundance of the SLR fold. This lack of data includes not only the discovery of biological functions, but experimental SLR protein structures alone and in complex with their biochemical targets (Urosev et al., 2013).

The *L. pneumophila* genome contains five open reading frames that encode SLR proteins (Newton et al., 2007), making this pathogen a powerful model to study the biochemistry of the SLR fold. These genes encode the SLR proteins LpnE, EnhC, LidL, Lpg1062, and Lpg1356. Lpg1356 was recently named LceB and will be referred to as such in this study (Wexler et al., 2022). The proteins are thought to be secreted virulence factors; however, the translocation mechanism of each remains unclear. To the best of our knowledge, LpnE, which contains a signal sequence, is neither translocated by the Type II secretion system (T2SS) or T4SS (Newton et al., 2007). There is data that shows LceB is translocated by the T4SS (Wexler et al., 2022), but LceB also contains a general N‐terminal secretory signal (sec‐signal) making the overall mechanism of its secretion unclear.

In terms of biological function, each of the five *L. pneumophila* SLR proteins has been studied. Both LpnE and EnhC have been extensively characterized and are known to assist in host cellular invasion (Cirillo et al., 2000; Newton et al., 2006). Additionally, these two proteins along with LidL are important in the signaling events required for proper trafficking of *L. pneumophilia* within macrophage cells (Conover et al., 2003; Newton et al., 2007). The protein Lpg1062 is required for growth in *Naegleria gruberi* (Park et al., 2020), is predicted to have eight SLR folds (Paysan‐Lafosse et al., 2023), and like LceB contains a sec‐signal sequence (Armenteros et al., 2019). LceB was recently discovered to be one of the nine core effectors in *Legionella* (Gomez‐Valero et al., 2019). Further investigation showed that a deletion mutant of LceB grew similarly to wildtype strains during competition assays using *Acanthamoeba castellanii* as a host (Wexler et al., 2022). Furthermore, an elegant study utilizing transposon mutagenesis also concluded that LceB was not important for intracellular growth *in A. castellanii*. This same study also determined that LceB was not required for growth in *Acanthamoeba polyphaga*, *Hartmannella vermiformis*, and *N. gruberi*, but necessary for growth in human U937 macrophages (Park et al., 2020). Thus, although the biochemical function of LceB remains unclear, its requirement for growth in macrophages suggests that it may play a specialized role in *Legionella* pathogenesis of human hosts.

Given that LceB is a secreted SLR protein effector of unknown function, we sought to characterize the structure of LceB to provide insight into its biochemical function at the molecular level. Here we solve an X‐ray crystal structure of LceB that reveals an SLR protein in agreement with bioinformatic predictions. Structural analysis of LceB compared to its close homologs reveal several conserved structural features and sequence motifs that provide insight into the role of LceB during *Legionella* infection. Furthermore, LceB crystallized with three copies in the asymmetric unit which adopted unique conformations different from the AlphaFold prediction. Overall, our data shows a dynamic SLR *Legionella* effector that contains several structural and sequence motifs also found in functionally characterized bacterial toxins.

## 2 RESULTS AND DISCUSSION

### 2.1 The overall structure of LceB

**FIGURE 1:**
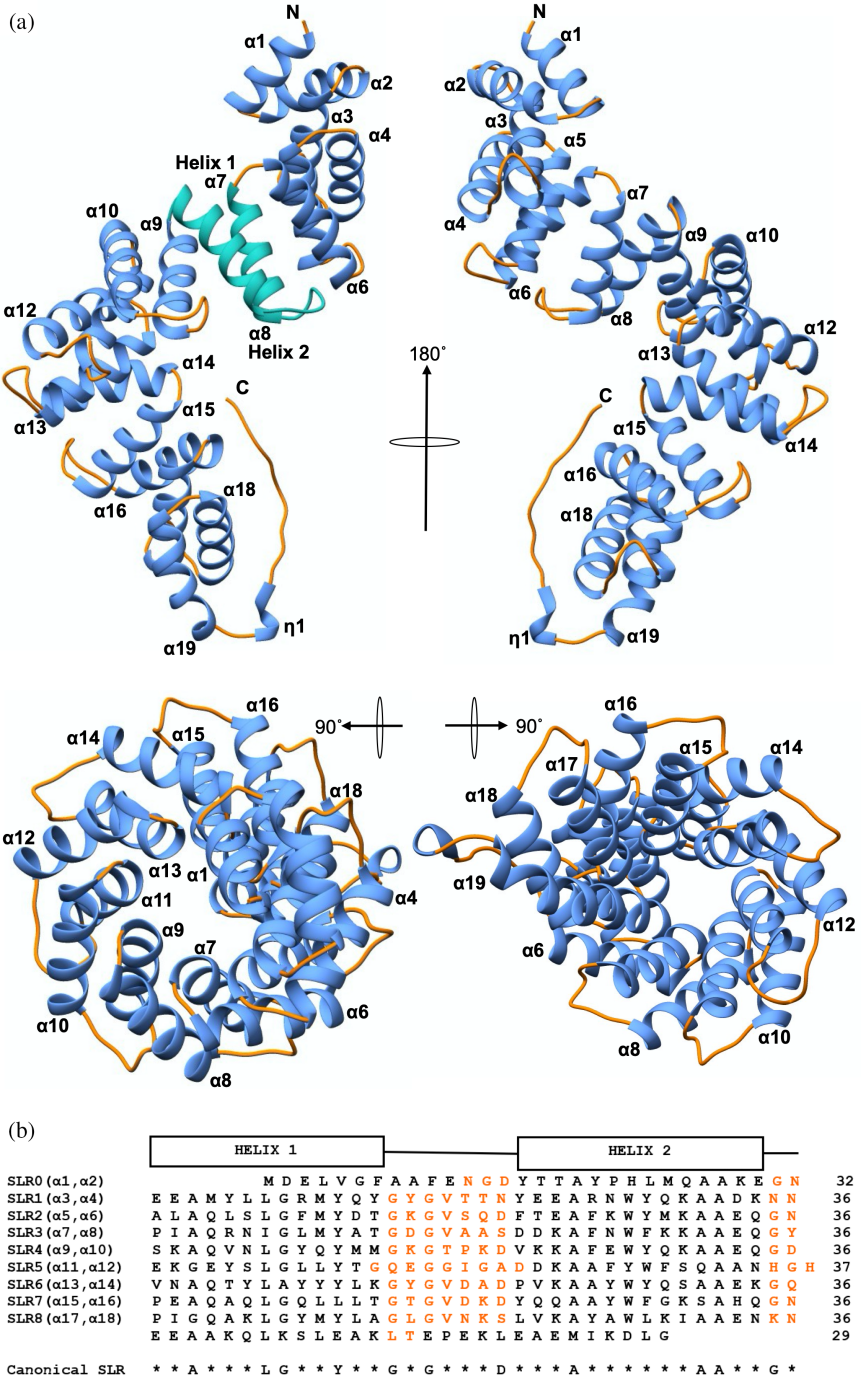
X‐ray crystal structure of LceB. (a) Four views of *Legionella pneumophila* LceB are shown as a ribbon diagram. The N and C terminus are labeled with a secondary structure indicated as alpha‐helices (*α*) and 3_10_ helices as (*η*). Helices are colored in blue or green and loop regions in orange. A single SLR made up of Helix 1 (α7) and Helix 2 (α8) is depicted in sea green. (b) Alignment of LceB SLR's. Secondary structure elements can be observed above the SLR sequences (Helix 1 and 2). The canonical SLR is located below the sequences with loop regions colored orange.

To purify LceB for crystallization, residues 1–18 were removed from the expression construct as they are predicted to be a sec‐signal (Armenteros et al., 2019). A full‐length LceB construct with an N‐terminal 6His‐tag was also created but failed to produce crystals. Instead, a construct spanning residues 22–366 from *L. pneumophila* containing an N‐terminal 6His‐tag was purified and screened for crystallization. Initially, poorly diffracting crystals were obtained. To improve the crystallization quality, the buffer for LceB was optimized using nanodifferential scanning fluorimetry (nanoDSF) by monitoring the change in tryptophan fluorescence during thermal denaturation (Figure S1 and Table S1). This resulted in a significant increase of the LceB *T*_m_ from ~63 to ~68°C by raising the NaCl concentration from 250 to 750 mM. LceB was repurified in the optimized buffer, crystallized, and an X‐ray crystal structure solved to a resolution of 2.7 Å (Figure 1 and Table 1). LceB crystallized in space group P3_2_ with three chains in the asymmetric unit (Figure S2). Analysis of the asymmetric unit by PDBePISA (Krissinel & Henrick, 2007) suggests that these are likely crystal packing artifacts as the contact surface for each chain varies with the largest interaction at 644.3 Å^2^ (Krissinel & Henrick, 2007) (Table S2).

**TABLE 1:** X‐ray data collection and refinement statistics.

Data collection	LceB
Wavelength (Å)	1.18082
Space group	P3_2_
Cell dimensions	
*a*, *b*, *c* (Å)	116. 79, 116.79, 88.35
*α*, *β*, *γ* (°)	90.0, 90.0, 120.0
Number of monomers in an asymmetric unit	3
Resolution (Å)	48.72–2.70 (2.80–2.70)
Total reflections	190,820 (23457)
Unique reflections	36,973 (3696)
CC (1/2)	0.999 (0.499)
*R*_merge_	0.021 (1.159)
*R*_pim_	0.011 (0.565)
*I*/*σI*	20.5 (2.69)
Completeness (%)	99.91 (100.0)
Multiplicity	4.7 (5.2)
Refinement	
*R*_work_/*R*_free_	0.2169/0.2509
Average *B*‐factors (Å^2^)	111.35
Protein	111.51
Ligands	99.98
Water	89.89
No. of atoms	8279
Protein	8207
Ligands	66
Water	46
RMS deviations	
Bond lengths (Å)	0.002
Bond angles (°)	0.38
Ramachandran plot (%)	
Total favored	93.67
Total allowed	6.14
PDB code	8SXQ

As shown in Figure 1, LceB is composed primarily of α‐helices forming a right‐handed superhelix that is ~93 Å long ending in an extended C‐terminal disordered region. There are 19 α‐helices which form eight pairs of antiparallel helices with each pair making up a SLR. A single SLR (SLR3) is highlighted in turquoise for clarity as helix 1 (α7) and helix 2 (α8) in Figure 1a. The sequences of each repeat unit are shown in Figure 1b. The concave inner surface of the LceB superhelix is composed of helix 1 and the convex outer surface is made of helix 2 of each SLR. Of note, is that the first two α‐helices (α1 and α2) at the N‐terminus of LceB are not composed of the required number of residues or adhere to the canonical SLR sequence. However, these N‐terminal helices conform to the SLR or TPR fold. Additionally, the C‐terminal helix (α19) appears to have no mate and we hypothesize that it acts as a capping helix to shelter the concave hydrophobic regions which are found in solenoid proteins like LceB (Mittl & Schneider‐Brachert, 2007). For example, in TPR proteins, capping helices are common and may help to increase protein stability and solubility (D'Andrea & Regan, 2003). Finally, LceB terminates in a 3_10_ helix that leads into a long‐extended tail.

The SLRs in LceB are separated from each other by two residues except for SLR5 and SLR6, where three residues make up the loop region (Figure 1b). Typically, the first residue of these loops are glycines, and are overall strictly conserved while the second residue is variable. Additionally, all the SLR inter‐helix loop regions in LceB are made up of seven residues except for SLR5 which contains nine. It is important to note that two definitions exist for describing SLR proteins (Letunic et al., 2021; Lüthy et al., 2004). The definition used in this paper is that from the Smart database (Letunic et al., 2021), where the length of the loop regions between helices within one SLR are long (4–12 residues) and the connecting regions between SLRs are short (three residues) (Mittl & Schneider‐Brachert, 2007). This was chosen as LceB conforms to the consensus sequence of SLRs described by the Smart database and not the other SLR description. In the alternative SLR description, the opposite is observed, in particular, shorter loop regions between helices and longer loops between SLRs (Luthy et al., 2002, 2004). Interestingly, the alternative definition is used in the structural characterization of LpnE in *L. pneumophila* (Voth et al., 2019).

### 2.2 Molecular surface properties of LceB

**FIGURE 2:**
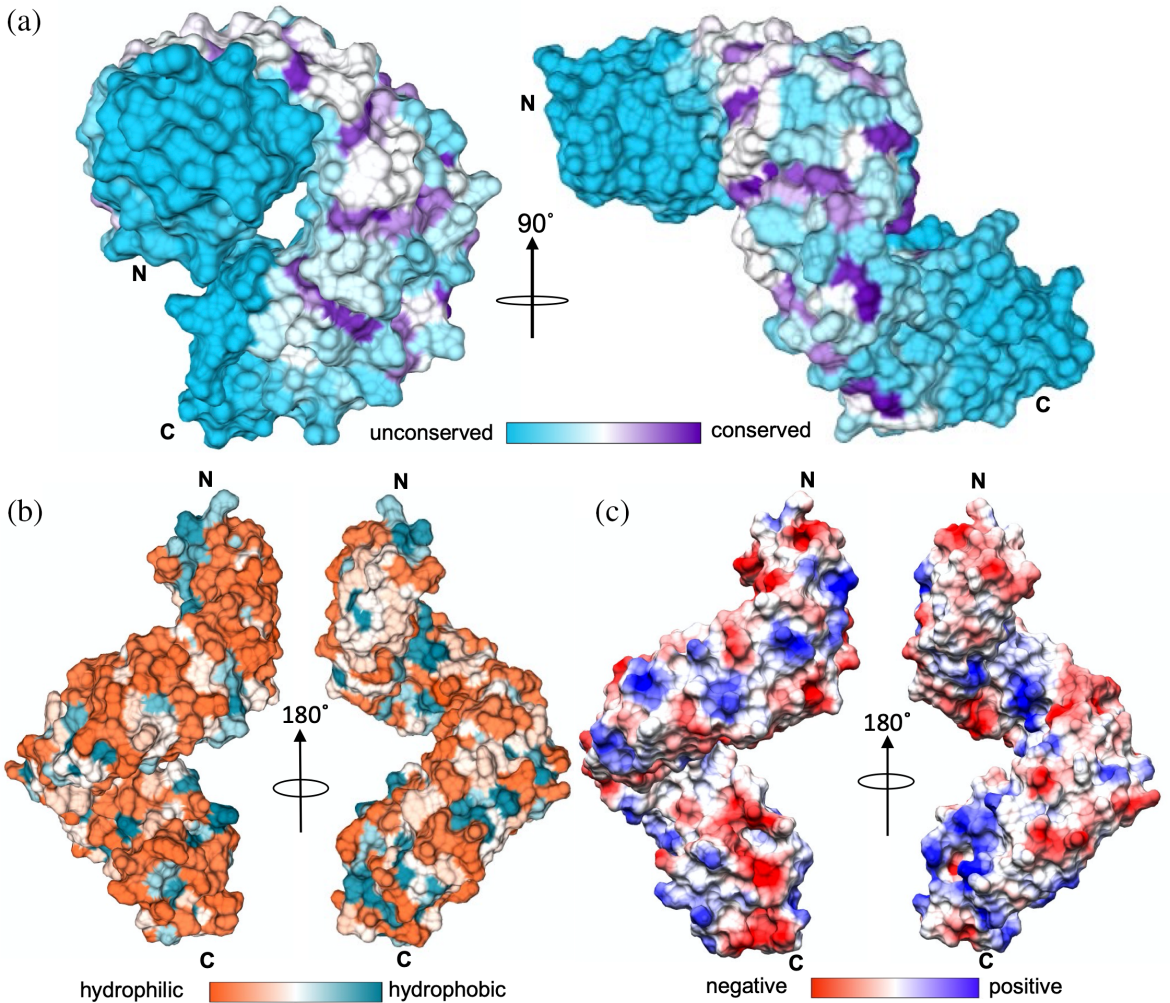
Molecular surface properties of LceB. (a) Residue surface conservation of LceB generated by Consurf (https://consurf.tau.ac.il/). Unconserved surfaces are colored in turquoise and conserved surfaces in deep purple. Areas of white or light shades indicate partial conservation or homologous residue regions. (b) Hydrophobic surface representation of LceB. A gradient of hydrophilic surfaces (orange) to hydrophobic patches (blue‐green) is drawn. (c) LceB colored by the Coulombic surface or electrostatic potential ranging from negative surface (red) to a positive surface (blue) as calculated by UCSF Chimera. In each panel, the N‐ and C‐termini of LceB are indicated.

To gain insight into a biochemical function for LceB, we first analyzed the structure for surface residue conservation for a potential active site or binding‐partner surface. As shown in Figure 2a, the majority of the LceB surface shows no residue conservation. Notably, the areas of most variability are at the N and C‐termini. This includes α1 and α2 that conform to the SLR fold but do not contain the SLR sequence, and the capping helix α19 with the extended C‐terminal tail. Additionally, no areas of residue conservation or predicted active site motifs (Yariv et al., 2023) are readily apparent on the concave surface of LceB. However, there are repeating patches of conservation along the entire length of the convex surface and defined stripes (Figure 2a). This contrasts with TPR proteins where the concave surface is commonly responsible for binding interactions (D'Andrea & Regan, 2003).

We next plotted both the hydrophobic and electrostatic properties of the LceB surface to observe if any of these properties align to the repeating patches of conservation in LceB (Figure 2b,c). As shown, the surface of LceB is very hydrophilic with few exposed nonpolar residues and no obvious hydrophobic patches (Figure 2b). The N‐terminal surface of LceB shows a slight overall negative charge while the C‐terminal convex region appears to have a positively charged patch (Figure 2c). As both the N‐ and C‐terminal regions show low residue conservation, it is unlikely that these electrostatics correlate with a conserved SLR‐domain function. Overall, neither hydrophobic regions or electrostatic regions aligned to areas of residue conservation in LceB (Figure 2). Regardless, as TPR family proteins are variable in sequence due to their unique functions (Buttner, 2008; Shanker et al., 2016) these observed molecular features could be important for biological partner interactions specific to LceB.

### 2.3 LceB contains motifs of known function

**FIGURE 3:**
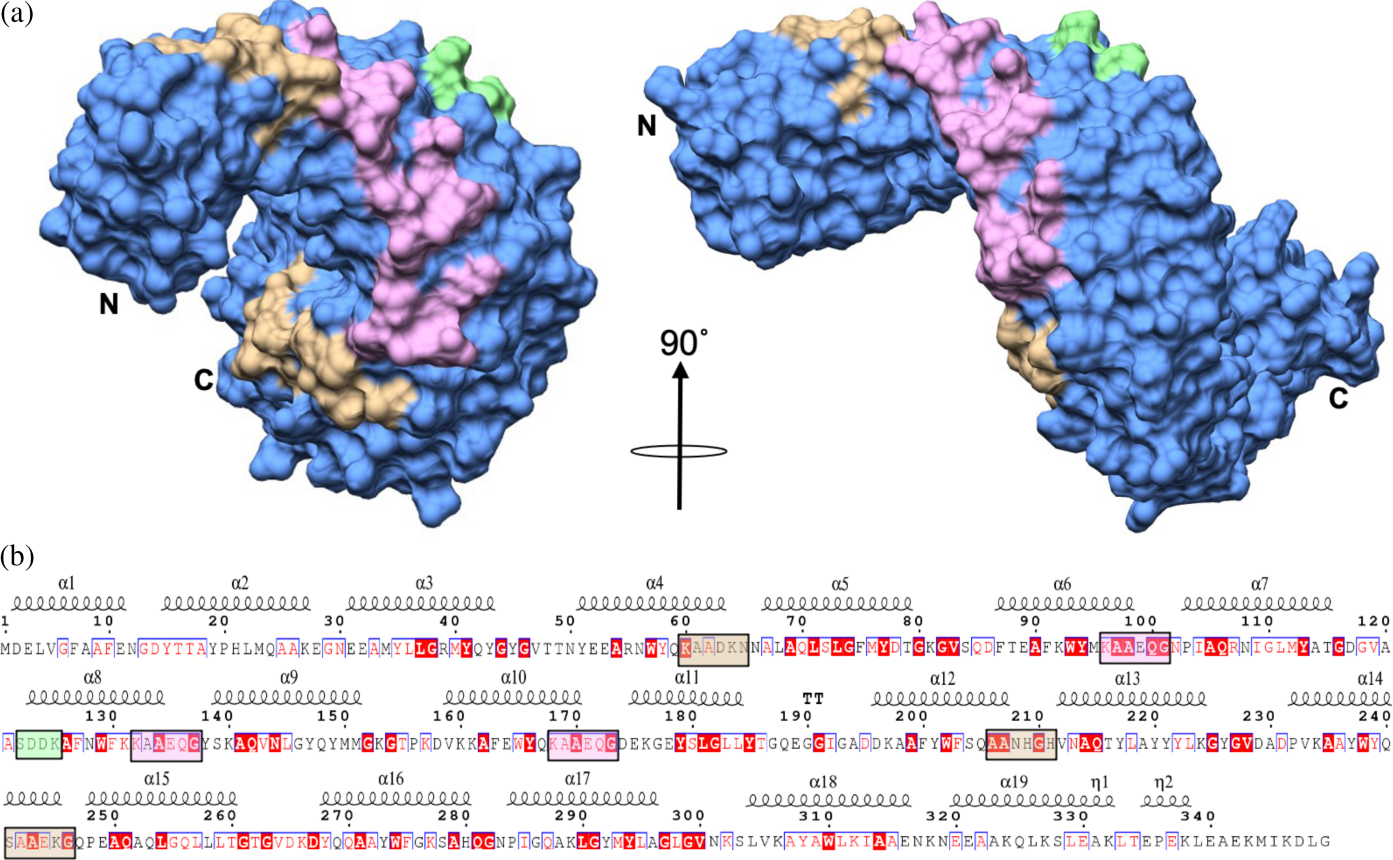
Conserved surface residue motifs of LceB. (a) Molecular surface representation of LceB showing predicted functional regions of LceB. The repeated KAAEQG sequence motif is highlighted in pink and is bounded by two closely related motifs in brown. A catalytic serine motif (SXXK) observed in known SLR β‐lactamases is colored green. (b) LceB amino acid sequence plotted with corresponding secondary structure and residue conservation using Espript (https://espript.ibcp.fr/). Residues highlighted in red indicate strict conservation, homologous residues are colored red, while unconserved residues are colored black.

LceB exhibits a repeated motif (KAAEQG) that is found in other SLR proteins as well as proteins that do not contain this fold. Intriguingly, the motif is not observed in the related TPR proteins to the best of our knowledge (de Castro et al., 2006). A search for LceB structural homologs using the Dali server (Holm, 2022) showed that the KAAEQG sequence and its variations are found in all the listed LceB structural relatives except for HcpC (Table 2). A biochemical function for this motif has not been assigned, but the motif has been observed in proteins with wide ranging functions, including bacterial cell division and purine biosynthesis (de Castro et al., 2006; Sayers et al., 2021). The KAAEQG motif in LceB is always within the second helix of the SLR and can be observed in SLR2 (α5‐6), SLR3 (α7‐8), and SLR4 (α9‐10) representing a partially conserved surface (pink) on LceB (Figures 1b and 3a). Importantly, the motif itself is highly conserved especially at the second alanine and terminal glycine residue. Substitution of other positions are always with a homologous residue. SLR1 (α3‐4), SLR5 (α11‐12), and SLR6 (α13‐14) contain variations of the KAAEQG motif and alternate with the conserved KAAEQG surface (brown, Figure 3a). Namely, SLR1 (α3‐4) has the terminal glycine replaced with lysine, and both SLR5 (α11‐12) and SLR6 (α13‐14) have the initial lysine substituted to serine (Figure 3b). In many SLR proteins, and observed in LceB, the conserved alanine and glycine residues allow tight packing of the repeats (Urosev et al., 2013). Additionally, this motif pattern is considered to be important to maintain the angular geometry between repeats, and thus the overall structure of the SLR fold (Urosev et al., 2013). However, as the KAAEQG and variant motifs also create a partially conserved surface found on the edge of the convex surface of LceB, this may indicate a potential binding site for a biological interaction partner.

**TABLE 2:** LceB structural homologs.

PDB id (protein name)	Bacterium	*Z*‐score	RMSD	% sequence identity	Function
4BWR (EsiB)	*Escherichia coli*	33.4	2.4	34	Inhibition of neutrophil chemotaxis and activation (Newton et al., 2006)
6OK3	*Oxalobacter formigenes*	33.2	2.8	34	Unknown (Sonn‐Segev et al., 2020)
6DEH (LpnE)	*Legionella pneumophila*	29.9	4.4	40	Host cell invasion, vacuole trafficking (Urosev et al., 2013)
6ONW	*Oxalobacter formigenes*	24.4	4.6	31	Unknown (Sonn‐Segev et al., 2020)
1OUV (HcpC)	*Helicobacter pylori*	23.9	4.5	27	Unknown (Sonn‐Segev et al., 2020)
6ORK	*Oxalobacter formingenes*	23.6	2.2	33	Unknown (Sonn‐Segev et al., 2020)

LceB also contains an SXXK motif on its surface depicted in green (Figure 3a). This motif is found in the structurally homologous protein HcpC from *H. pylori* (Table 2). HcpC is from a family of *Helicobacter* cysteine‐rich proteins (Hcps) found in epsilon proteobacteria (Lüthy et al., 2004) which includes HcpA, B, D, and E also found in *H. pylori* (Lüthy et al., 2004; Mittl & Schneider‐Brachert, 2007). Members of this family of proteins have been shown to bind and weakly hydrolyze derivatives of penicillin (Luthy et al., 2002; Mittl et al., 2000). The active site in β‐lactamases contain a catalytic serine within a SXXK motif where the lysine is invariable and two other motifs, ((S/Y)X(N/S/D/C)) and ((K/L)(T/S)G) separated by approximately 70 residues (Massova & Mobashery, 1998; Mittl et al., 2000). Interestingly, HcpA contains only the SXXK and ((S/Y)X(N/S/D/C)) motifs, yet retains hydrolysis activity (Mittl et al., 2000). Additionally, HcpB was shown to co‐purify with *N*‐acetylmuramic acid and has all three motifs (Luthy et al., 2002). Overall, these results suggested that Hcps may be involved in cell‐wall biosynthesis (Luthy et al., 2002, 2004). Given that LceB has a sec‐signal and contains a SXXK motif, it is tempting to speculate that it may have a dual function in the periplasm or is also part of the T4SS apparatus. However, this is a small motif that requires pairing with other motifs for β‐lactamase function. As such, it is unclear if the SXXK motif has any functional significance for LceB.

**FIGURE 4:**
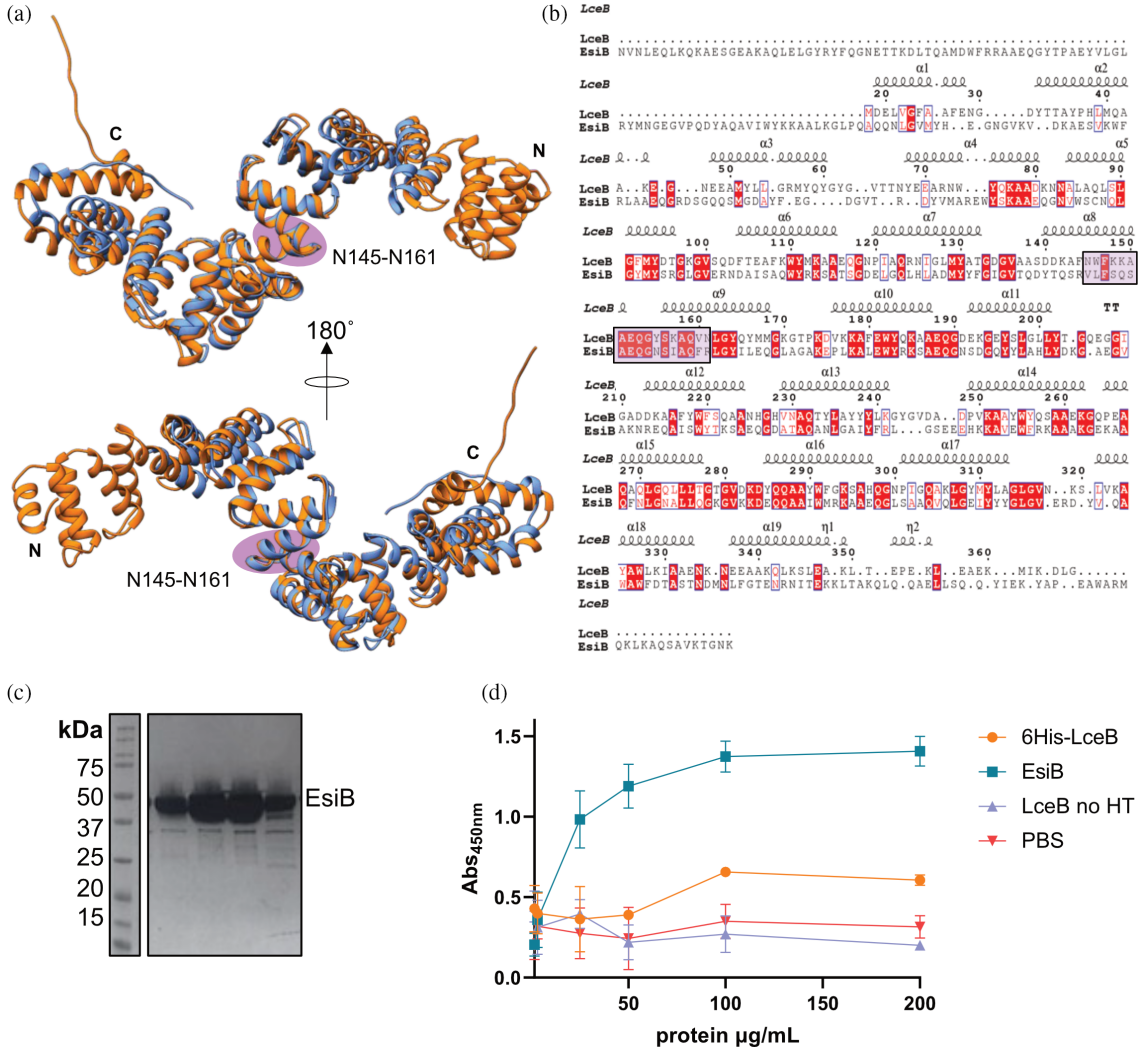
Structural alignment of LceB with EsiB from *E. coli* (a) LceB (cornflower blue) aligned with EsiB (PDB 4BWR) (orange) is shown in two views with a 180° rotation. (b) The sequence alignment based on the structural alignment of LceB and EsiB from the Dali server is shown. The secondary structural elements and residue conservation is plotted by Espript (https://espript.ibcp.fr/). Residues highlighted in red indicate strict conservation, homologous residues are colored red, while unconserved residues are not bolded and colored black. The sequence that EsiB uses to bind SIgA is highlighted with a purple oval on the structures in panel (a) and by a box on the sequence in panel (b). This corresponds to a KAAEQG sequence motif. (c) Coomassie stained SDS‐PAGE gel showing the purity of EsiB after purification by SEC. (d) ELISA of protein binding to SIgA. 6His‐LceB (orange), EsiB (blue), LceB no HT (purple) and PBS (pink). Interaction with SIgA was measured by increasing protein concentrations and blotting with anti‐His conjugated HRP antibody. The HRP enzyme product absorbs at 450 nm after quenching the reaction. The graph was plotted using Graph Pad Prism version 9.4.1.

The closest known structural homolog to LceB is the protein EsiB (Table 2 and Figure 4a). As shown in Figure 4a, LceB aligns extremely well to the core of EsiB but with variations at each terminus. Namely, EsiB has three additional SLRs at the N‐terminus and the packing of the C‐terminal domain varies. Similar to LceB, EsiB also contains a capping helix at the C‐terminus and follows the same SLR definition as LceB. Again, this definition is that each SLR consists of two α‐helices linked by seven amino acids with three residues connecting adjacent SLRs (Urosev et al., 2013). EsiB is a virulence factor found in uropathogenic *Escherichia coli* (UPEC), a subtype of extraintestinal pathogenic *E.coli* (ExPEC), CFT073 *E. coli* (Urosev et al., 2013). ExPEC is pathogenic to both humans and animals causing neonatal meningitis (NMEC), septicemia as well as urinary tract infections (UTIs) (Moriel et al., 2010). Mucosal surfaces in humans are protected considerably by secretory immunoglobulin A (SIgA) which is hydrophilic and negatively charged (Pastorello et al., 2013). SIgA binds to the FcαRI receptor along with macrophage 1 antigen (Mac‐1, CD11b/CD18) (van Gool & van Egmond, 2021). However, this binding is sterically hindered due to the secretory component (SC) of SIgA but still leads to the activation of neutrophils and destruction of the pathogen (Breedveld & van Egmond, 2019; Pastorello et al., 2013; van Gool & van Egmond, 2021). EsiB functions by binding to SIgA without blocking its interaction with the FcαRI receptor. Overall, this results in the failure of the neutrophil to be activated, aiding in the survival of the pathogen (Pastorello et al., 2013). Moreover, the biochemical activity of EsiB hinders chemotaxis of neutrophils (Pastorello et al., 2013). The specific amino acid sequence in EsiB that interacts with SIgA is 244‐VLFS*QSAEQG*NSIAQFR‐260 (Urosev et al., 2013). Variations of this sequence can be observed throughout both EsiB and LceB (Figure 3b, Figure 4b). Specifically, the SIgA binding motif includes the KAAEQG repeat motif of LceB (underlined in the SIgA binding sequence). This close structural and sequence motif identity of LceB to EsiB could indicate that the role of LceB in the LCV is to inhibit an immune response.

To test this hypothesis, an enzyme‐linked immunosorbent assay (ELISA) adapted from Pasterollo et al. and Ausubel et al. (Ausubel et al., 2002; Pastorello et al., 2013). was performed. In this assay, we used EsiB as a positive control and both PBS and LceB without a 6His‐tag (LceB no HT) as negative controls. First, we designed a 6His‐tag EsiB construct, which we could produce at high‐yield and purity (Figure 4c). As expected, EsiB bound to SIgA in a dose dependent manner (Figure 4d, blue). Additionally, both PBS alone and LceB no HT only showed a background level of binding and no dose dependent increase of absorbance (Figure 4d, pink and purple). Interestingly, 6His‐LceB showed a low level of interaction above the background that seemed to reach saturation at 100 μg/mL (Figure 4d, orange). It appears that LceB can interact with SIgA, but the binding is significantly weaker than EsiB. Furthermore, we hypothesize that the weak interaction is most likely nonspecific. This is due to 6His‐LceB binding levels saturating at an absorbance just over the background (see PBS and LceB no HT), while never reaching an absorbance comparable to EsiB. Either LceB requires additional components to interact with SIgA, or alternatively, LceB has a different biological interaction partner.

### 2.4 Structural comparison of LceB to known SLR proteins

In addition to HcpC and EsiB, LceB has several additional structural homologs as determined by the Dali server (Holm, 2022). The top six nonredundant structural hits had *Z*‐scores ranging from 33.4 to 23.6 with the remaining hits all scoring below a *Z*‐score of 20 (Table 2). The RMSD and sequence identity for the top six hits ranged from 2.2 to 4.6 Å^2^ and 27%–40%, respectively. Intriguingly, three of the six hits (PDBid: 6OK3, 6ONW, and 6ORK) are from *Oxalobacter formigenes*, a major degrader of oxalate in the human gut (Allison et al., 1985), but the function of each of these proteins has not yet been determined. However, the predicted annotation for each of these gene products is beta‐lactamase and carbon‐nitrogen bond hydrolase activity.

LceB is also structurally related to LpnE (PDBid 6DEH) from *L. pneumophila* (Holm, 2022). LpnE is a well characterized SLR family protein that has both known protein and lipid binding partners. *In vitro*, LpnE binds the immunoglobulin‐type folds of Obscurin‐like protein 1 (OBSL1), Oculocerebrorenal syndrome of Lowe protein (OCRL), and the lipid phosphatidylinositol‐3‐phosphate (PI3P) (Newton et al., 2007; Weber et al., 2009). These binding activities occur within the LCV during host invasion (Newton et al., 2007; Weber et al., 2009). OCRL dephosphorylates phosphatidylinositol‐4,5‐bisphosphate (PtdIns(4,5)P_2_) and has been shown to impede *Legionella* infection (Berman et al., 2000; Weber et al., 2009; Zhang et al., 1995). LpnE has previously been shown to be important in trafficking of vacuoles and host cell invasion, an activity that appears to require all eight of its SLR motifs (Newton et al., 2007). It is important to note that two of the other five known *Legionella* SLR proteins LidL and EnhC are also involved in vacuolar trafficking (Cirillo et al., 2000; Conover et al., 2003; Newton et al., 2006; Newton et al., 2007). For example, EnhC is able to complement an LpnE mutant strain, and LpnE is able to complement an EnhC deficient strain (Bandyopadhyay et al., 2012). This common phenotype, coupled with the fact that Dot/Icm effectors are redundant (Luo & Isberg, 2004) further suggests that LceB may aid in maintaining the LCV within human macrophages.

Several general structural similarities also exist when LceB is compared to other SLR proteins. Like LceB, many SLR proteins contain secretion and signal sequences (EsiB, EnhC, HcpC, LpnE). For example, the N‐terminal signal sequence (1–21) of LpnE is responsible for localization to the *cis*‐Golgi in HEK293 cells and without the sequence, LpnE is found to be retained within the host cytosol (Voth et al., 2019). Given this, it is likely that LceB requires a specific localization to exert its biological function. The LceB structural homologs span a repeat range of 7–21 SLRs and homologs such as EsiB, HcpC, and LpnE also exhibit a C‐terminal capping helix that serves to stabilize the overall curved SLR structure (Lüthy et al., 2004; Newton et al., 2007; Urosev et al., 2013). Furthermore, many residues are conserved for structural purposes in the SLR proteins. Alanines in positions 3 and 32 (LceB numbering) are highly conserved within each SLR which allows tight packing of the repeats (Urosev et al., 2013) (Figures 1b and 3b). Specifically, the conserved alanine at the C‐terminus of an SLR associates with another conserved alanine at the N‐terminal of a subsequent repeat to properly pack the repeats together. Additionally, the tryptophan at position 27 (LceB numbering) although not part of the canonical SLR sequence is conserved in nearly all the SLR homologs listed in Table 2 and makes stabilizing contacts with the previous SLR in the sequence. The pattern for the residue associations are also seen in EsiB where the authors hypothesized that this interaction was important for maintaining the angular geometry between repeats (Urosev et al., 2013).

### 2.5 LceB shows structural flexibility

**FIGURE 5:**
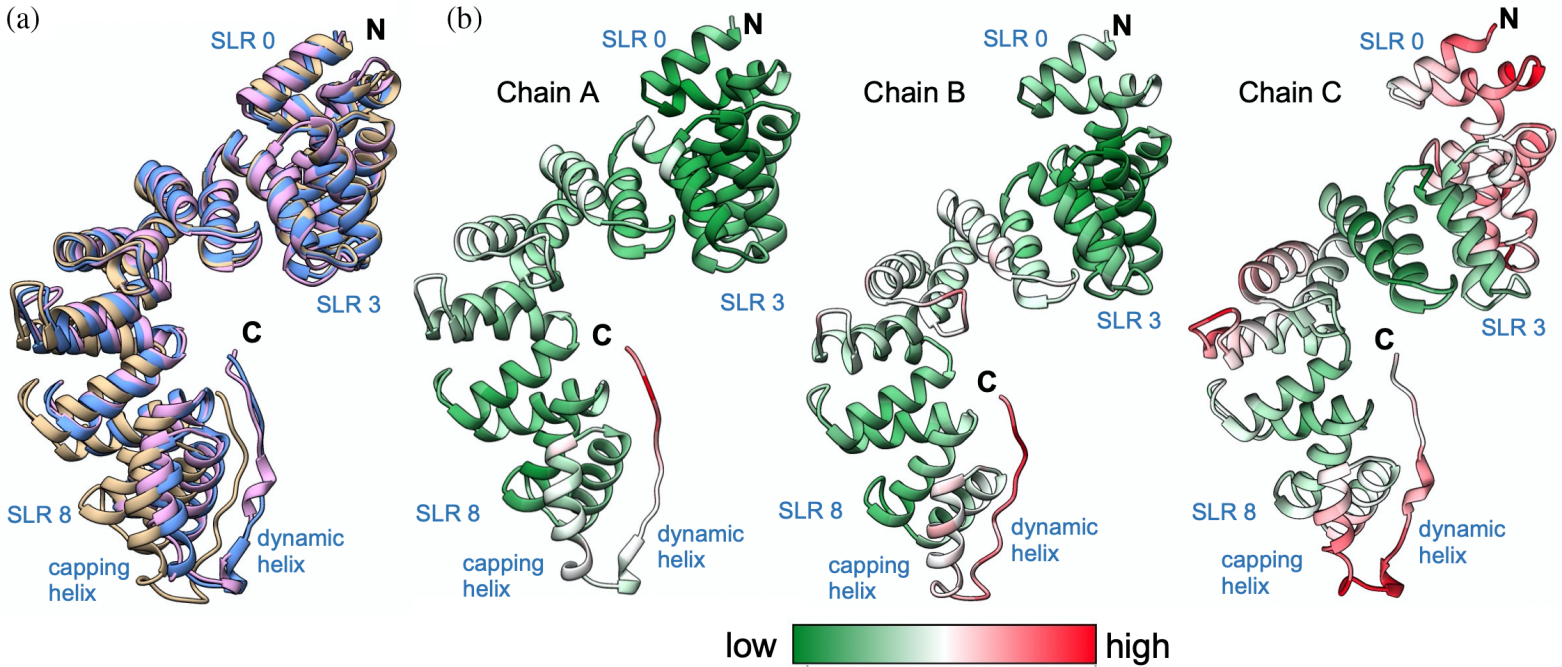
Comparison of the three LceB copies in the asymmetric unit. (a) Superimposition of LceB chains. Chain A is in cornflower blue, Chain B is in tan, and Chain C is in plum. All panels have the N and C termini labeled. (b) For each chain the observed B‐factors are colored by gradient from low (green) to high (red). Each chain has the N‐ and C‐terminus indicated with relevant structural features highlighted.

The three chains of LceB in the asymmetric unit (Figure S2) were captured in different conformations within the crystal (Figure 5a). Chain A superimposes on Chain B with an RMSD of 0.8 Å^2^ across 198 equivalent C‐alpha atoms (2.6 Å^2^ for all 348 residues) and Chain C of 0.9 Å^2^ for 325 Cα atoms (1.2 Å^2^ for all). Chain B and C superimpose with an RMSD of 0.9 Å^2^ (168 Cα atoms) and 3.4 Å^2^ for all atoms. Between the three chains, the greatest variation was seen in both the N‐terminal (SLRs 0 to 2 or helices α1–α6) and C‐terminal regions (SLR8 or helices α17–α18). This includes the C‐terminal residues that appear to adopt a poorly resolved helix that ends in an extended conformation (Figure S3). Overall, it appears that LceB can “flex” to further open the inner concave surface as the C‐terminus of Chain B is pushed ~6 Å out relative to Chain A (Figure 5a).

**FIGURE 6:**
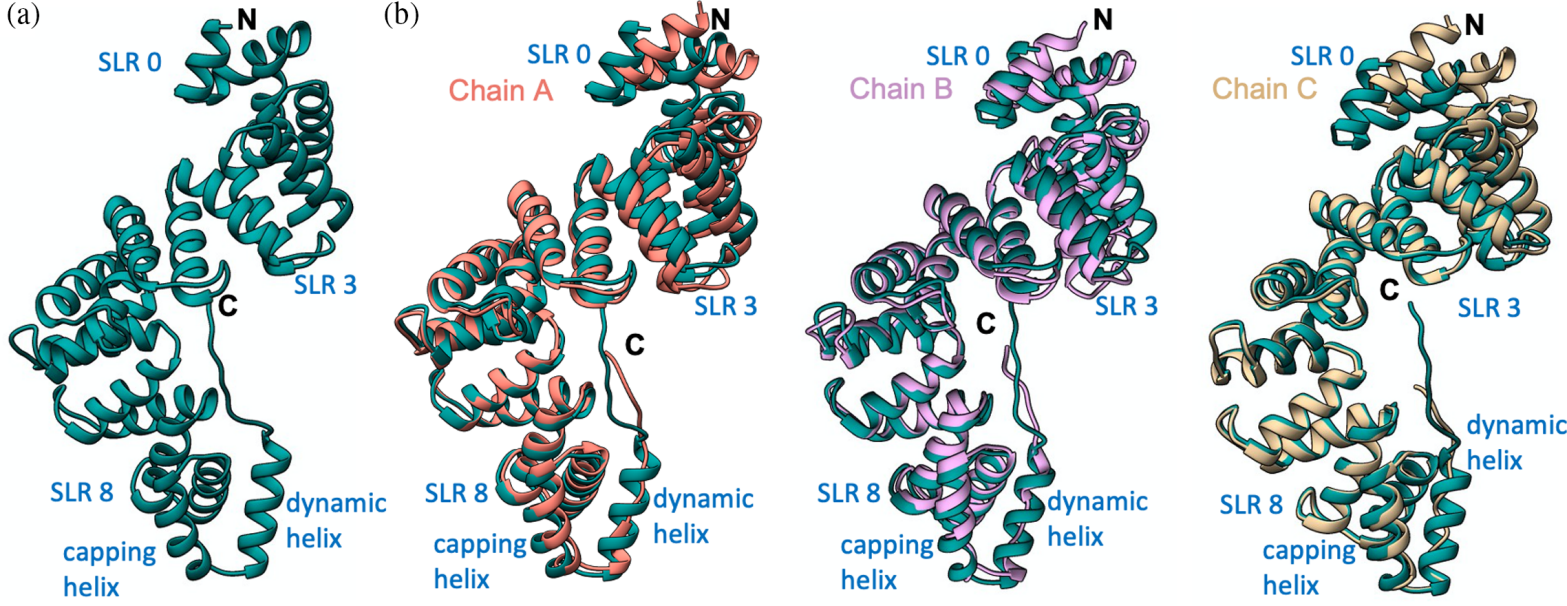
The crystal structure of LceB varies from the AlphaFold model. (a) LceB structure predicted by AlphaFold and used as a molecular replacement model. (b) The Alphafold model (steel blue) aligned with each chain in the LceB x‐ray crystal structure. Chain A (cornflower blue), chain B (plum), and chain C (tan). Each chain has the N and C terminus indicated with relevant structural features highlighted.

Plotting the B‐factors for each chain in the LceB crystal structure reveals varying levels of motion across the protein. Chain A showed the least motion followed by chain B with chain C having the highest overall thermal parameters (Figure 5b). In chain C, both the N and C‐terminal regions had the highest observed B‐factors suggesting these regions of LceB may be flexible relative to the rest of the protein. Although the thermal parameters could relate to crystal packing artifacts, it must be noted that in all three chains the C‐terminal region was exposed to solvent and had poor electron density (Figure S3). The density appears to suggest an α‐helix, and in fact, the AlphaFold model used for molecular replacement predicts the C‐terminus as a long α‐helix (Figure 6a). Taken together, the experimental data and the predictive model indicate that the C‐terminal region of LceB is likely a conformationally dynamic helix. Given that this last helix is beyond the capping helix (α19), sequence variable, and not a canonical SLR mate, the structural and biochemical role of this region is unclear.

Further supporting the experimental data that LceB is conformationally dynamic, the AlphaFold model predicts a different overall conformation for LceB as compared to the X‐ray crystal structure. In addition to showing a folded C‐terminal helix, the packing arrangement of SLRs 0 to 3 in the AlphaFold model is bent so as to close the concave surface (Figure 6b). For both chain A and chain C, SLR0 (α1 and α2) is moved about 4.5 Å outwards relative to the predicted structure with differences in the position of helices averaging ~4 Å in the entire N‐terminal half of LceB. The conformational change is even more pronounced when compared to chain B. Helices α1–α5, or SLRs 0–1 and half of SLR 2, in the experimental structure are more than 7 Å and up to just over ~8 Å shifted outward relative to the AlphaFold model. When comparing the predicted model and the three LceB chains of the experimental structure, we observe a closed conformation in the AlphaFold model and an open conformation in chain B, with both chain A and C roughly at the midpoint between the extremes.

### 2.6 LceB oligomerizes in solution

LceB was readily purified by metal chelating affinity chromatography followed by size exclusion chromatography (SEC) where it was observed to elute as at least two distinct peaks (Figure S4A). Although no obvious dimer or larger oligomer was apparent from the asymmetric unit in the crystal structure (Figure S2), this suggests that LceB may be in equilibrium between a monomer and higher‐order oligomer. Alternatively, the two peaks could represent a compact and elongated conformation of LceB as suggested by the observed flexibility and dynamic C‐terminus (Figures 5 and 6).

**FIGURE 7:**
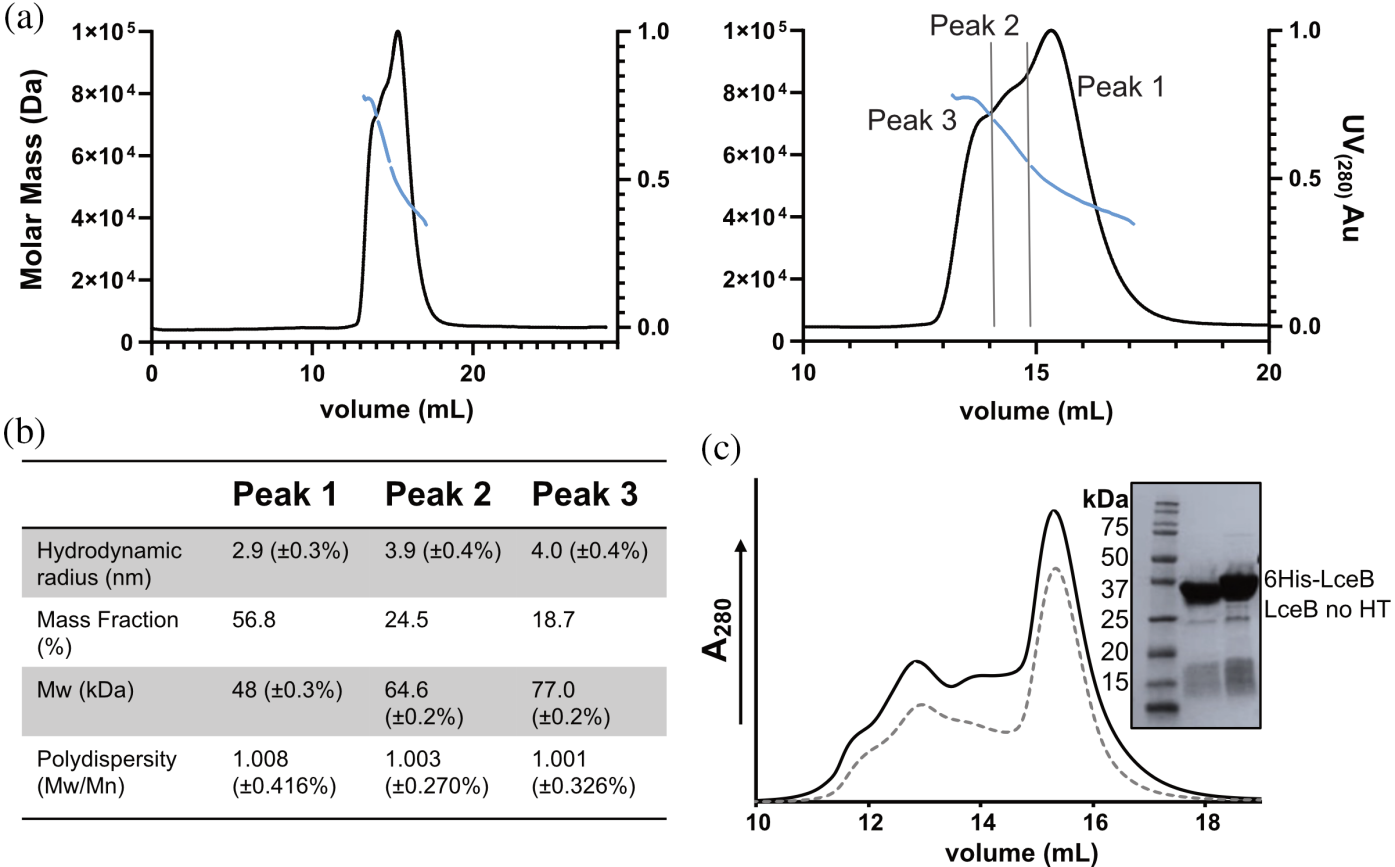
Solution state analysis of LceB. (a) SEC‐MALS trace of LceB showing calculated molecular mass fit of the peak(s) in blue (left). Zoom‐in of SEC‐MALS trace showing three peaks (right). The boundaries separating the overlapping peaks for MALS fit are shown as gray lines. (b) Table of mass‐calculations from ASTRA analysis software. (c) SEC traces of 6His‐LceB (black line) and LceB no HT without an N‐terminal 6His‐tag (gray dotted line) are shown. Inset is a Coomassie stained SDS‐PAGE gel showing the purity of the LceB SEC samples before loading. Graphs were plotted using GraphPad Prism version 9.4.1.

To better determine the oligomeric state of LceB, all SEC fractions (Figure S4A) were pooled together and reconcentrated for analysis by SEC coupled with multi‐angle light scattering (SEC‐MALS). In this experiment, LceB eluted as three peaks (Figure 7a). Peak one yielded a measured mass of 48 kDa and made up more than half of the mass fraction (56.8%) (Figure 7b). This peak likely corresponds to a monomeric species as the predicted molecular mass of an LceB monomer is 42 kDa. Peaks two and three yielded molecular masses and mass fractions of 64.6 kDa (24.5%) and 77 kDa (18.7%), respectively. Neither of these two measured molecular masses are enough to match that of a dimer (96 kDa) in this experiment, and together compose less than half of the protein sample. As SEC‐MALS should measure an absolute mass, it is possible that each peak is contaminated with higher or lower‐order oligomers as the tails from each peak lead into the next making precise size analysis by SEC‐MALS challenging.

In agreement with our observations, a study by Voth and colleagues in 2019 investigating the LceB homolog LpnE found similar behavior in solution (Voth et al., 2019). When SEC was performed with His‐tagged LpnE, the protein also eluted in two peaks. However, when SEC‐MALS was used to study an untagged version of LpnE, the protein eluted as a monomer. This suggested that the His‐tag was responsible for the self‐association of LpnE (Voth et al., 2019). Given this similarity, we expected the larger particle size peaks observed for LceB could also be due to cloning artifacts. To address this possibility, we removed the N‐terminal 6His‐tag by digestion with thrombin (Figure S4B) and performed SEC analysis of 6His‐LceB and digested LceB at the same concentration (Figure 7c). As shown in the chromatograms, the removal of the affinity tag (LceB no HT) resulted in a nearly identical elution profile to 6His‐LceB. Furthermore, the SDS‐PAGEs gels of both experiments show that the major protein in each SEC fraction is LceB (Figure S4C,D). Either with or without the 6His‐tag, LceB appears to oligomerize in solution but the exact nature of the LceB oligomer is unknown from these experiments.

As SEC and SEC‐MALS require relatively high protein concentrations and proteins sometimes nonspecifically interact with gel‐filtration matrices, we could not be certain if our observations were due to either factor. To address these concerns and to better determine the exact nature of the LceB oligomers, we measured the oligomeric state of 6His‐LceB and LceB no HT in solution by mass photometry. For these experiments, we also included the full length LceB construct. Mass photometry can accurately measure particle size in solution by observing single molecule (or particle) scattering events at low concentrations (nanomolar). This can often better resolve the properties of different particles in a heterogeneous solution and removes effects due to high protein concentrations (Sonn‐Segev et al., 2020). As mass photometry requires very little sample and data can be obtained within minutes, it also allowed us efficiently and rapidly test LceB with or without a 6His‐tag in both physiological and high‐salt buffer conditions, and the full length LceB construct.

**FIGURE 8:**
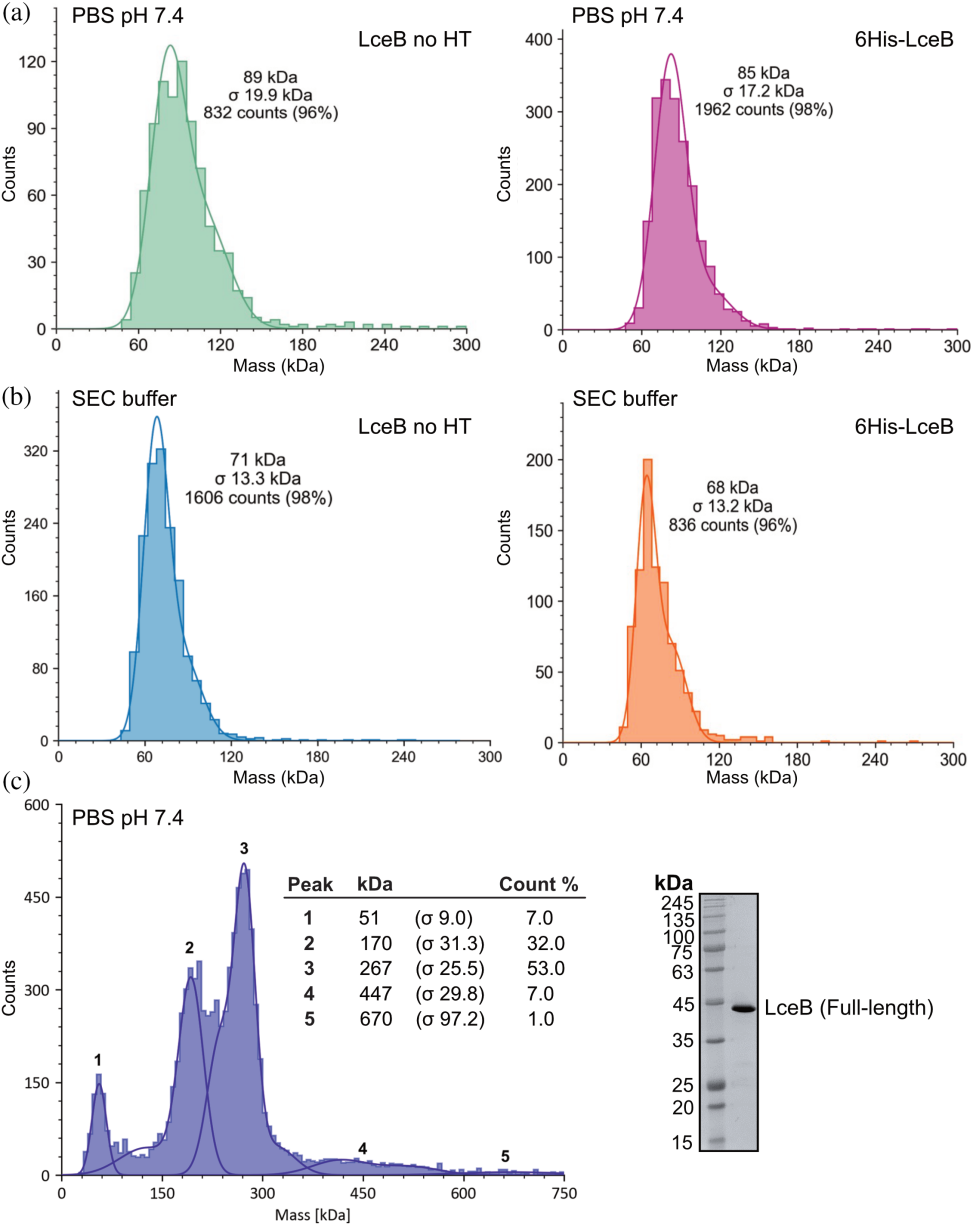
LceB forms higher‐order oligomers in solution. Molecular weight histograms obtained from mass photometry measurements of purified LceB. (a) LceB (22–366) without a 6His‐tag (LceB no HT) and with the cloning artifact (6His‐LceB) in PBS pH 7.4. (b) LceB (22–366) without a 6His‐tag (LceB no HT) and with the cloning artifact (6His‐LceB) in SEC buffer (Tris pH 7.5 750 mM NaCl). (c) Molecular weight histogram obtained from mass photometry measurements of purified full‐length LceB in PBS pH 7.4 (left). Approximate oligomeric states are Peak 1 = monomer, Peak 2 = trimer, Peak 3 = pentamer or hexamer, Peak 4 = decamer, and Peak 5 = unknown multimer. Right: Coomassie stained SDS‐PAGE gel of full‐length LceB used in mass‐photometry experiments. The peak and standard deviation (*σ*) of calculated molecular weights are shown.

In our experiments, 6His‐LceB and LceB no HT both showed a similar behavior in physiological conditions (phosphate buffered saline, PBS pH 7.4) (Figure 8a) as they did in the high salt SEC buffer (Tris pH 7.5 750 mM NaCl) (Figure 8b and Figure S1). Again, this demonstrates that the 6His‐tag is not significantly influencing the oligomeric state of LceB. However, upon comparing the mass distributions in PBS to the high‐salt SEC buffer we observe less counts at higher molecular weights and a sharper, less disperse peak (Figure 8a,b). This could suggest an electrostatic component to LceB oligomerization or that LceB self‐association is nonspecific. Overall, the results in PBS indicate that the major species of LceB is a dimer (~90 kDa ± 20). The error in fit is likely due to a skew from overlapping monomeric and higher‐order oligomers (Figure 8a). Likewise, the high salt SEC buffer appears to compete with LceB self‐association increasing the amount of monomer present and skewing the fit to a lower overall molecular weight (~70 ± 13 kDa) (Figure 8b).

Contrary to our experiments with the truncated LceB crystallization construct, experiments with full length 6His‐LceB revealed two major peaks corresponding to the estimated MW of a trimer (peak 2) and pentamer (peak 3) (Figure 8c). Oligomer sizes were estimated from the full‐length LceB monomer appearing to be 51 kDa in solution (peak 1). These states accounted for over 80% of the analyzed sample. Additionally, minor species corresponding to a monomer (peak 1), decamer (peak 4), and a possible 12 to 14‐mer (peak 5) of LceB were detected. Although given the broadness of peak 4 and the low counts of peak 5, these may simply be nonspecific aggregates or contamination. Unexpectedly, the N‐terminal 21 amino acids of LceB appear to encourage oligomerization into a trimer or pentamer. Without the N‐terminus LceB instead favors a monomer–dimer equilibrium (Figure 7 and Figure 8). We must note that each sample had to be measured at a different concentration (0.15–5 nM) due to signal intensity, with full‐length LceB at the highest concentration. However, given the extremely low concentrations used, and that mass‐photometry measures single particles, we do not expect that the concentration difference has significantly influenced the experiment. Specifically, even with the difference between the truncated and full‐length LceB samples, the overall dilute concentrations of both samples should prevent the appearance of nonspecific protein aggregates.

It is tempting to speculate that the three copies of LceB in the asymmetric unit may be reflective of the solution‐state trimer, or at least provides insight into how LceB may self‐associate (Figure S2). Additionally, we were curious as to how the N‐terminus might contribute to oligomerization, as full‐length LceB does form trimers (Figure 8c). To address these questions, we attempted to model an LceB dimer and an LceB trimer using AlphaFold (Jumper et al., 2021) (Figure S5) with both LceB (22–366) and full‐length LceB. For all runs no prediction was similar to the asymmetric unit in the crystal (Figure S2) which supports the data from PDBePISA (Table S2). However, the predicted alignment error (PAE) plots for both the dimer (Figure S5A) and the trimers (Figure S5B,C) showed very little confidence (all errors greater than 5 Å) making the predictions unreliable.

## 3 CONCLUSION

An X‐ray crystal structure of LceB from the respiratory pathogen *Legionella pneumophila* has been solved to a resolution of 2.7 Å in the space group P3_2._ LceB crystallized as three super‐helical chains in the asymmetric unit, with each chain composed of 8 SLRs (Figure 1). Upon structural analysis we observed that LceB contains a repeated motif, KAAEQG that is also found in its closest structural homologs (Figure 3). Although this motif has a structural role (Urosev et al., 2013), it may also be functionally important. Namely, the closest structural homolog to LceB, the protein EsiB in uropathogenic *E. coli,* requires this motif to bind the host protein SIgA (Figure 4 and Table 2). The binding of EsiB to SIgA interferes with the host response inhibiting neutrophil activation, leading to pathogen survival (Pastorello et al., 2013). It is therefore likely that this repeated conserved motif sequence in LceB is also the site of protein binding, especially as it forms a conserved surface on LceB (Figures 2a and 3a). However, a protein binding partner for LceB has yet to be determined as our results demonstrate SIgA is likely not the primary interaction partner for LceB (Figure 4d). Additionally, LceB is structurally similar to another *Legionella* SLR protein, LpnE, which functions in vacuolar trafficking during host cell invasion. Taken together, this may be suggestive that LceB has a role in immune cell evasion and could be involved in a variety of other protein–protein interactions. Interestingly, both these proteins have a general secretory signal which at least for LpnE, is thought to be necessary for its localization within a host. This raises the question of why LceB contains a sec‐signal, especially as data exists that it is secreted by the sec‐independent T4SS (Wexler et al., 2022). Addressing this, our data does show that the N‐terminal sec‐signal region of LceB is important for oligomerization in solution (Figure 8). However, the mechanism of how the N‐terminus promotes LceB oligomerization or its biological implications are currently unknown.

Our structural data also indicates that LceB is conformationally dynamic, or at the very least flexible in solution. The chains in the asymmetric unit showed different conformations, with chain B showing the most variation from chain A and chain C (Figure 5a). Furthermore, the AlphaFold model of LceB also predicted a significantly different conformation from those observed in the crystal structure, especially in the N‐terminus the protein (Figure 6). This, combined with the high B‐factors and poor density for the putative C‐terminal helix, strongly indicates that LceB is conformationally dynamic in solution. Moreover, LceB readily forms oligomers in solution (Figure 8) which could indicate it has adhesin‐like biological properties to form large complexes with its eukaryotic host target. For example, the *Legionella* SLR effector LpnE is known to form oligomers that interact with its binding partner OCRL (Voth et al., 2019). Overall, our determination of an experimental LceB structure has allowed the exploration of its molecular surface properties and demonstrated a level of conformational flexibility that may shed light not only into its biological function, but also into the structure and biochemistry of the SLR family of proteins. The molecular data of LceB presented here will lead to a better understanding of *Legionella* effector activities and host pathogen interactions.

## 4 MATERIALS AND METHODS

### 4.1 Protein expression and purification of LceB

LceB (*Legionella pneumophila* Philadelphia‐1, Uniprot accession number Q5ZVT4) codon optimized for *E. coli* was obtained in pET22b vector from Genscript. LceB without the predicted secretion signal was PCR amplified off the plasmid to include restriction sites NdeI and BamHI, and subcloned into pET15b‐Amp to generate LceB (residues 22–366) with an N‐terminal 6His‐tag in *E. coli* DH5α. Once the expression construct was generated, the vector was transformed into *E. coli* BL21 (DE3) Gold cells. The cells were then grown in Lysogeny Broth (LB) at 37°C until an Optical Density (OD) of 0.6 at 600 nm absorbance was reached. IPTG was added to the culture at a final concentration of 1 mM to induce protein expression for ~20 h at 20°C. After incubation, the culture was spun down at 4200 *g* for 30 min and resuspended in wash buffer (50 mM Tris pH 7.5, 500 or 750 mM NaCl, 25 mM imidazole). PMSF and MgCl_2_ were added at final concentrations of 1 mM and 10 mM respectively, along with a small amount of DNase. An Emulsiflex‐C3 High Pressure Homogenizer (Avestin) was employed for cell lysis with the lysate subsequently subjected to centrifugation at 17,000 *g* for 30 min at 4°C. The supernatant was added to a wash buffer‐equilibrated nickel‐NTA affinity gravity flow column (GoldBio) and elution of the protein was achieved using 1 column volume of elution buffer (50 mM Tris pH 7.5, 500 or 750 mM NaCl, 500 mM imidazole). The eluate was concentrated using a 10 kDa concentrator (Sigma) and further purified by SEC using a Superdex 75 (16/600) HiLoad column (Cytiva) equilibrated in gel filtration buffer (50 mM Tris pH 7.5, 250 mM or 750 mM NaCl). Protein was further purified by ion exchange chromatography (IEX) after nickel‐NTA and SEC. LceB in gel filtration buffer (750 mM NaCl) was diluted to 25 mM NaCl and then eluted by increasing NaCl concentration (0–300 mM) at pH 7.5 50 mM Tris on an anion exchange (Q‐sepharose) column (Cytiva). Fractions from SEC and IEX were run on an 8%–12% gradient SDS‐PAGE and dyed with Coomassie Brilliant Blue to determine fractions which contained pure protein. Selected fractions were concentrated as previously for crystallization experiments.

For purification of full‐length LceB, *lceB* was cloned into the vector pMCSG682BPTEV to include an N‐terminal 6His‐tag with TEV cleavage site. Full‐length LceB was purified by metal affinity chromatography similar to the truncated form of LceB but required the inclusion of 5% glycerol at high‐concentrations. After purification by SEC, full‐length LceB was dialyzed O/N into phosphate‐buffered saline (PBS) without glycerol.

### 4.2 Protein expression and purification of EsiB

EsiB (residues 24 to 490) from uropathogenic *E. coli* (*c5321* gene) was obtained from Twist Bioscience. The EsiB construct was cloned into pET29b using restriction sites NdeI and XhoI to contain a C‐terminal 6His‐tag. After successful transformation into *E.coli* BL21(DE3) Gold cells, the bacteria was grown in LB to an OD of 0.6 at and induced overnight at 20°C. Cells were harvested, lysed, and purified by metal affinity chromatography as previously stated for LceB. After, purified EsiB was concentrated and loaded onto a Superdex 75 (16/600) HiLoad column (Cytiva) for further purification and buffer exchanged into PBS pH 7.4. Fractions from SEC were run on an 8%–12% gradient SDS‐PAGE and dyed with Coomassie Brilliant Blue (BioRad) to determine which fractions contained pure protein. Selected fractions were concentrated and used for ELISAs.

### 4.3 Nano‐differential scanning fluorimetry (nanoDSF)

Thermal denaturation of LceB (22–366) was completed using a Prometheus NT.48 (NanoTemper). Purified protein in 50 mM Tris pH 7.5 250 mM NaCl was diluted seven‐fold to a final concentration of 50 μM into Tris and HEPES buffer systems and 25‐fold to a final concentration of 10 μM in Bis‐Tris buffer systems. Both the pH and salt concentrations were varied. Buffer systems used were: HEPES pH 7.5 and 8, Tris pH 7.5 and 8, Bis‐Tris pH 6.5 and 7, sodium acetate pH 3.8 and 4.5. Salt concentrations employed were 0, 50, 100, 200, 400 and 750 mM NaCl. Samples were then subjected to thermal denaturation by heating from 20 to 95°C at a rate of 1.0°C/min. The fluorescence intensity was monitored at 330 and 350 nm after excitation of tryptophan at 280 nm. Melting temperatures (*T*_m_) were calculated from the first derivative of the 350/330 nm fluorescence emission ratio. All samples in the sodium acetate buffer systems showed immediate visible precipitation upon addition of LceB and were not included in the nanoDSF experiment.

### 4.4 Protein crystallization

Crystallization of purified LceB (22–366) was screened for utilizing commercially available screens (NeXtal) and a Crystal Gryphon robot (Art Robbins Instruments). Crystals of LceB grew in gel filtration buffer and 0.1 M Citric Acid pH 4.0, 10% MPD in a 1:1 drop ratio at 15 and 10 mg/mL of protein at 4°C. These conditions were optimized using the sitting drop vapor diffusion method at 4°C (moved to 20°C 5 days later), with crystals generated in varying concentrations of citric acid (0.04 M‐0.15 M) pH 4.0 and MPD (8%–12%) (mother liquor) at a concentration of 10 mg/mL. As the optimized crystals were of insufficient size, they were micro‐seeded using the SeedBead kit (Hampton Research). Crystals were obtained in gel filtration buffer 50 mM Tris 750 mM NaCl pH 7.5 at 20°C from 0.1 M citric acid pH 4.0, 10% MPD at a concentration of 7 mg/mL and used for subsequent data collection and refinement.

### 4.5 Data collection and refinement

The dataset for LceB was collected at the Canadian Light Source beamline CMCF‐BM (08B1). Protein crystals were cryo‐protected stepwise in 15%, 25% and finally 30% MPD before flash freezing directly in liquid nitrogen. Data was processed using XDS (Kabsch, 2010) and CCP4 (Winn et al., 2011). Initial phases were obtained with Phenix (Adams et al., 2010) by molecular replacement utilizing a model of LceB predicted by AlphaFold (Jumper et al., 2021). The protein was built in Coot (Emsley et al., 2010) and refined with Phenix (Adams et al., 2010) Refmac5 (Murshudov et al., 1997) and TLS (Painter & Merritt, 2006). UCSF Chimera (Pettersen et al., 2004) and GraphPad Prism version 9.4.1 were utilized for molecular graphics.

### 4.6 Enzyme‐linked immunosorbent assay (ELISA)

Prior to performing the ELISA adapted from Pasterollo et al. and Ausubel et al. (Ausubel et al., 2002; Pastorello et al., 2013), all proteins (6His‐LceB and LceB no HT (LceB no His‐tag) (22–366) and EsiB) were dialyzed O/N into PBS pH 7.4 if they were not already in this buffer. 6His‐tagged EsiB was used as positive control, and negative controls consisted of untagged LceB and PBS pH 7.4. The coating antibody (human colostrum SIgA) (Sigma‐Aldrich) was diluted in PBS for a final concentration of 1 μg/mL. A volume of 50 μL of this solution was added to each well and incubated overnight at 4°C with gentle rotation. The next day, wells were washed three times with PBS pH 7.4 and was blocked using blocking buffer (PBS pH 7.4 0.05% BSA 0.25% Tween). Plates were then incubated at 37°C for 2 h with rotation and washed three times as mentioned above. The antigen (6His‐LceB, LceB, and EsiB) was used at the following concentrations: 200, 100, 50, 25, 3, and 1.5 μg/mL. After another 2 h of incubation at 37°C with gentle rotation, the wells were washed three times with PBS. Anti‐His‐Tag mouse mAb (HRP conjugate) (Cell Signaling Technology) was diluted 1:500 and incubated for 2 h at room temperature. Wells were washed again with PBS after incubation and the substrate solution 1‐Step™ Turbo TMB‐ELISA was added to each well for 10 min. After this time, 12.5% H_2_SO_4_ was added in an equal volume to stop the reaction. The absorbance was read at 450 nM with a Synergy HTX (BioTek) plate reader. Reads were obtained from three replicates. The averages were plotted along with standard deviation using GraphPad Prism.

### 4.7 SEC coupled multiple angle light scattering (SEC‐MALS)

Purified LceB (22–366) was concentrated to 15 mg/mL in 50 mM TRIS pH 7.5 750 mM NaCl and spun in a 0.1 μM filter to remove aggregates before SEC‐MALS analysis. SEC‐MALS data were collected on a DAWN HELEOS II detector (Wyatt Technology) coupled to an AKTA Pure (Cytiva) with an in‐line UV cell (Cytiva). For this experiment a Superdex 200 (10/300) increase column (Cytiva) was used. LceB was injected after the system was equilibrated in gel filtration buffer and detectors aligned and normalized with a 15 mg/mL BSA control (Sigma). All experiments were performed at 25°C. Analysis of the data was completed using ASTRA analysis software (Wyatt Technologies).

### 4.8 SEC of digested LceB

Purified LceB (22–366) was dialyzed O/N at 4°C with the addition of 1:200 mg/mg thrombin:LceB (Sigma) to remove the N‐terminal 6His‐tag. After digestion, LceB was first centrifuged to remove precipitation and the soluble fraction was re‐purified over an NTA‐agarose affinity column to remove undigested material. The flow‐through of unbound digested‐LceB was concentrated and then analyzed by SEC using an SD200 increase 10/300 column with an AKTAgo (Cytiva) in the optimized buffer (50 mM Tris pH 7.5 750 mM NaCl). Both undigested and digested LceB were analyzed at 3 mg/mL.

### 4.9 Mass photometry of LceB

Mass photometry experiments for 6His‐LceB (22–366), LceB (22–366), and LceB full‐length were performed using a Refeyn One Mass Photometer machine and AcquireMP 2023.1.1 software (Refeyn Ltd). 6His‐LceB and LceB were analyzed in PBS pH 7.4 and 50 mM Tris 750 mM NaCl pH 7.5, full‐length LceB was analyzed in PBS pH 7.4. Measurements were taken in 3 mm × 1 mm silicone gaskets (Grace Bio‐Labs) and 1.5H high‐precision coverslip glass (24 × 50 mm, Deckgläser and Thorlabs). Briefly, coverslips were sonicated for 10 min in Milli‐Q water, then isopropanol, and then Milli‐Q water again. Slides were dried under air stream. The calibration curve was generated using bovine serum albumin (BSA), alcohol dehydrogenase, beta‐amylase, apoferritin and thyroglobulin in both PBS pH 7.4 and 50 mM Tris 750 mM NaCl pH 7.5. Protein samples were added to gaskets filled with the appropriate buffer and were diluted to protein concentrations ranging from 0.13 to 5 nM to optimize signal. Final concentrations used were 6His‐LceB (0.15 nM), LceB no HT (0.12 nM), and LceB full length was 5 nM. Higher concentrations of either LceB (22–366) construct saturated the detector. Images were acquired for 1–2 min depending on the sample with a 331 or 477 nm laser. DiscoverMP software 2023.1.2 (Refeyn Ltd) was used to visualize and analyze the measurements.

## AUTHOR CONTRIBUTIONS

**Gerd Prehna:** Conceptualization; funding acquisition; writing – original draft; writing – review and editing; visualization; project administration; resources; supervision. **Tiffany V. Penner:** Methodology; investigation; writing – original draft; writing – review and editing; conceptualization; validation; visualization. **Neil Lorente Cobo:** Conceptualization; methodology; investigation; writing – original draft; writing – review and editing. **Deepak T. Patel:** Investigation; methodology. **Dhruvin Patel:** Investigation; methodology. **Alexei Savchenko:** Writing – original draft; funding acquisition; resources; supervision; writing – review and editing. **Ann Karen C. Brassinga:** Funding acquisition; writing – original draft; supervision; resources; writing – review and editing.

## FUNDING INFORMATION

This work was supported by the Natural Sciences and Engineering Research Council of Canada (NSERC) grants RGPIN‐2017‐04878 to AS, RGPIN‐2019‐05490 to AKB, and RGPIN‐2018‐04968 to GP, and a Canadian Foundation for Innovation award (CFI) 37841 to GP. This work was also supported by a University of Manitoba Research Grants Program (URGP) to AKB and GP.

## CONFLICT OF INTEREST STATEMENT

The authors declare that they have no conflicts of interest with the contents of this article.

## Supporting information


**Data S1.** Supporting Information.

## Data Availability

The x‐ray structure and diffraction data reported in this paper are deposited in the Protein Data Bank under the accession code 8SXQ.
